# Can Land Transfer Alleviate the Poverty of the Elderly? Evidence from Rural China

**DOI:** 10.3390/ijerph182111288

**Published:** 2021-10-27

**Authors:** Wei Wang, Xin Luo, Chongmei Zhang, Jiahao Song, Dingde Xu

**Affiliations:** 1 Department of Agriculture and Forestry Economics and Management, College of Management, Sichuan Agricultural University, 211 Huimin Rd, Chengdu 130062, China; wangwei@sicau.edu.cn (W.W.); 2020306028@stu.sicau.edu.cn (X.L.); 2020209054@stu.sicau.edu.cn (C.Z.); 2 Department of Rural and Regional Development, College of Management, Sichuan Agricultural University, 211 Huimin Rd, Chengdu 130062, China; 3 Sichuan Center for Rural Development Research, College of Management, Sichuan Agricultural University, 211 Huimin Rd, Chengdu 130062, China

**Keywords:** agricultural land transfer, elderly poverty, relative poverty, PSM

## Abstract

This study explores the impact of farmland transfer on the multidimensional relative poverty of the elderly in rural areas to provide a reference for the study of rural land transfer in China and improve the welfare system for the elderly. Based on the China Family Panel Studies (CFPS) rural sample data in 2018, this paper uses the AF multidimensional index measurement method to assess multidimensional relative poverty in rural areas. Logit regression estimation examines the single index poverty of rural older adults transferred from rural land and the impact of multidimensional relative poverty, using the propensity score matching method (PSM) to analyze the results’ robustness. The transfer of agricultural land has different impacts on the poverty of different rural elderly poverty indicators and negatively affects the comprehensive effect of rural elderly poverty. The transfer of agricultural land significantly alleviates rural elderly poverty. Reasonable and effective transfer of agricultural land, together with improved rural social security and a caring service system for the elderly, will promote the continuous operation of large-scale agricultural operations and alleviate rural elderly poverty.

## 1. Introduction

Over the past 40 years of reform and opening-up, China has accomplished world-renowned achievements in anti-poverty [1]. Measured by the current rural poverty standard at the current price, poverty dropped from an incidence of 97.5% at the end of 1978 to 0.6% at the end of 2019 [2]. By the end of 2020, all rural poor people in China will no longer experience poverty under the current poverty standards, and all impoverished counties will be stripped of their hats [3,4]. However, the resolution of absolute poverty does not mean the end of poverty alleviation. After 2020, the focus of poverty alleviation will shift from eliminating absolute poverty to alleviating relative poverty [5]. The goal of poverty alleviation will shift from “two no worries and three guarantees” to response and relief to avoid the unbalanced and inadequate development of multidimensional relative poverty transition [6,7]. It can be seen that establishing multidimensional relative poverty standards, identifying new poor people, establishing new poverty alleviation targets, and formulating new poverty reduction measures will become the new focus of academic research. These features are all based on multidimensional relative poverty measurements. Previously, poverty measurement was often based on the family [8,9], and family members were in the same poverty state. However, different family members may also have different poverty situations due to differences in close relationships, resource endowments, and decision-making power. Notably, the elderly from non-poor families may also be in a state of poverty [10]. According to the law of individual life cycle development [11,12], with increasing age, the elderly’s body functions become weaker, the probability of disease will increase correspondingly, and health status will decline, and the occurrence of labor migration of children and grandchildren has formed a unique rural elderly population [13]. The physical and psychological characteristics of the child’s labor force are reducing, the income level is declining, and the income structure is single, making it more vulnerable to the impact of poverty [14]. At the same time, the elderly among family members may be in a relatively weak position, with an increased risk of falling into relative poverty [15].Therefore, it is of great practical significance to separate the old age stage in the life cycle and study the problem of rural old age poverty. Due to the weakening of labor ability, it is difficult to go out to work. The income structure of the rural elderly is relatively simple, derived primarily from agricultural income, pensions and children’s intergenerational support, and other transferred income [16]. Current rural social security is still on China’s social security system margins [17]. The security system is imperfect, and the labor migration of children and grandchildren and the current secularization and rational value drive may affect intergenerational support, so agricultural income has become a more stable income mode for the rural elderly [18,19].

“Agriculture for the elderly” is an integral part of China’s small-scale peasant economy, and it is also the epitome of China’s agriculture and rural society [20]. The absence of the young and middle-aged within the leading agricultural business makes the rural elderly have to care for the contracted land [21,22]. This model has two sides. On the one hand, it can provide the elderly with a stable rations supply and agricultural income. However, on the other hand, the elderly with weakened physical functions may increase their labor burden and cause physical and psychological negatives, resulting in multidimensional poverty [23].

With the acceleration of the national agricultural modernization construction, fragmented land management can no longer meet the development needs of agricultural modernization, and land transfer is imperative [24]. In this context, many scholars have put forward new ideas of “land for security” and land rent for old-age care, advocating for “land for security” to enable the rural elderly to obtain fundamental old-age care and medical security [25]. Alternatively, using land rent to improve quality of life reduces the risk of the rural elderly falling into poverty [26,27]. So, can the transfer of agricultural land now alleviate the poverty of the elderly in rural areas? Clarifying this issue can provide a realistic basis for alleviating rural elderly poverty, providing theoretical references for rural land transfer and promoting an appropriate scale of agriculture. Based on the above, this article will discuss the impact of the transfer of agricultural land on the relative poverty of the elderly in rural areas, assess the outcome of agricultural land transfer on the rural elderly poverty, and research the issue of rural land transfer in China and welfare of the elderly. Overall, improvements to the system provide a reference for the best outcomes for the rural elderly.

## 2. Theoretical Analysis

**Figure 1:**
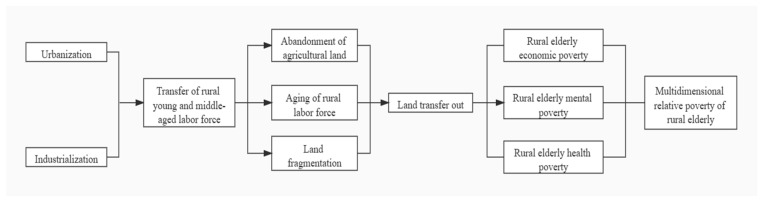
Theoretical analysis framework.

Changing the pattern of small-scale farming operations in rural areas and establishing modern agriculture with moderate scale operations has become the fundamental strategy for China’s agricultural modernization, national food security, and increasing farmers’ income [28]. At present, there are still two major obstacles in the process of agricultural modernization in China: one is the fragmentation of land; the other is the aging of the labor force [29]. As an effective way to solve land fragmentation and realize large-scale operations, the land transfer will promote agricultural modernization and may also impact rural elderly poverty [30,31]. Economic level, quality of life, and emotional status are the core content of poverty for the elderly. Traditional Chinese rural family care for the elderly often uses economic support, life care, and emotional comfort to reduce the possibility of the elderly falling into poverty [32]. The impact of the transfer of agricultural land on rural elderly poverty should also be considered from the three aspects of the economy, mental health, overall health, and wellbeing (see Figure 1).

One is the impact of the transfer of agricultural land on the economic poverty of the rural elderly. During industrialization and urbanization, China has faced many problems such as the massive transfer of rural labor to cities, the unsustainable old-age security of traditional families, and the severe poverty of the elderly in rural areas [33]. The rural social old-age security system is currently weaker than the city’s, both in absolute and relative terms. Coupled with the family pension instability caused by the outflow of children and decreased family size, the rural elderly have greater pension security. The degree depends on land income, and land is the last welfare guarantee [34,35]. As rural elderly age, their physical functions age and weaken, their labor capacity decreases, and the labor income obtained from the land decreases [36]. Some scholars believe that land transfer can highlight the property income function of land while retaining the transfer income of agricultural land subsidy for the elderly, which is a crucial strategy for increasing land income [37,38,39]. In contrast, other scholars argue that the economic impact of agricultural land on the elderly, in addition to the economic gain from the sale of agricultural products, can secure food rations. The transfer of land, then, removes the ability to provide food rations for themselves, increasing the burden of consumption expenditures such as food and affecting the living standards of the rural elderly [40].

Secondly, the role of agricultural land transfer in the spiritual poverty of the rural elderly also must be considered. The land has always played a vital role in the lives of rural China elderly, not only as a source of economic income but also as spiritual support and an essential carrier of “security” for the elderly farmers [41]. The outflow of children and the smaller rural families have affected the elderly emotional support. The hollowing out of rural areas and the weakness of grassroots organizations have also resulted in a lack of spiritual and recreational activities and emotional attention in the community [42]. The dual consideration of labor burden and farmland transfer rent may force the rural elderly to transfer their farmland, thus leading to a decrease in the happiness of the rural elderly and an increase in clinical depression levels [43]. The transfer of farmland may also reduce the frequency and length of return of outgoing laborers, affecting the mental poverty of rural elderly people. When the farmland is transferred, children no longer have to think about agricultural production and devote more time and energy to non-agricultural work, thus reducing the frequency and length of home visits. However, contrary to the above view, the transfer of agricultural land may also alleviate the mental poverty of the rural elderly, primarily because after the agricultural land transfer, the income of the rural elderly increases. The labor burden is reduced, and the health conditions improve. The rural elderly may invest more time, energy, and money participating in social activities organized by neighbors or communities to alleviate mental anguish, loneliness, and other emotions.

Finally, the role of agricultural land transfer on rural elderly health poverty will be considered. As the direct object of agricultural production activities, the land is a fundamental source of livelihood for farmers and an essential supplement to the rural social security system. Farmers rely on land production for their old-age needs. However, as they become older, their labor capacity decreases, the quality of agricultural production decreases, and output decreases. At the same time, the exodus of children hinders the inheritance of responsibility for farm work. Land becomes a burden for the elderly in rural areas and may become abandoned [44]. Land transfer can promote the moderate scale operation of agriculture and introduce the property function of agricultural land through the transfer of land management rights, replacing the income from land production with property income from renting out the right to use contracted land [45]. Following land transfer and income substitution, the labor burden of the rural elderly is also relieved to a greater extent. It may even have a positive impact on the physical health of the rural elderly. However, the transfer of agricultural land may also negatively impact the physical health of the rural elderly, mainly because the transfer of agricultural land can cause decreased happiness of the rural elderly and generate mental poverty through the development of clinical depression, which moderates physical health due to mental negativity.

Overall, the impact of agricultural land transfer on rural old-age poverty is complex, having a positive or negative impact on the same poverty dimension, and may also have different impacts on other dimensions of poverty. Therefore, it is necessary to select a composite poverty measure suitable for the post-2020 poverty standard to examine the comprehensive impact of the agricultural land transfer on rural old-age poverty. Multidimensional relative poverty covers the consideration of multidimensional poverty and adapts to the reality of China’s transition from solving absolute poverty to alleviate relative poverty in the post-2020 period. Consequently, this paper proposes two research hypotheses.

**Hypothesis** **1** **(H1).** *The transfer of agricultural land may have different effects on different dimensional indicators of old-age poverty.*

**Hypothesis** **2** **(H2).** *The combined effect of farmland transfer on rural old-age poverty is negative, i.e., farmland transfer can alleviate the occurrence of multidimensional relative poverty in rural old age.*

While considering both these research hypotheses, this paper will first investigate whether the transfer of agricultural land will have a two-way impact on the same dimension of poverty among rural elderly. Additionally, any different impacts of land transfer on different dimensions of poverty will be analyzed while constructing a multidimensional relative poverty system for rural elderly citizens. The basis of, then, validating the multidimensional relative poverty measures is using a Logit regression model. The propensity score matching method (PSM) was also used to test the robustness of the regression results to ensure the authenticity of the model estimates.

## 3. Research Design

### 3.1. Research Approach

The methodology was based on Alkire and Foster’s [46] Multidimensional Poverty Index measure using poverty deprivation counts [46]. Set each rural elderly individual surveyed in a different dimension as $y_{ij}$. $y_{ij}$ denotes the value taken by each individual i in the dimension j, i=1,2,3…n; j=1,2,3…d and forms an n×d dimensional matrix as shown in Equation (1):

(1) $$Y=\left(\begin{array}{ccc} y_{11} & \cdots & y_{1d}\\ \vdots & \ddots & \vdots \\ y_{n1} & \cdots & y_{nd} \end{array}\right)$$

Define a deprivation threshold for each dimension of poverty identification. $z_{j}(z_{j}<0)$ denotes the deprivation line in the $j^{th}$ dimension. For matrix Y, the deprivation matrix can be obtained as:

(2) $$g_{ij}=\{\begin{array}{l} 1,\;x_{ij}<z_{j}\\ 0,\;else \end{array}$$

$g_{ij}$ denotes the poverty status of individual i in dimension j. Additionally, define a column vector $c_{i}=[g_{ij}]$, that represents the total number of deprivation dimensions assumed by the $i^{th}$ older individual.

To sum up the dimensions, we need to consider the weight of each dimension, and in this paper, we choose the equal weight method, which is commonly used in this research field [46]. Specifically, define w to denote the weighted row vector of dimension d. Its element $W_{j}(W_{j}\in W)$ is the weight of dimension j, and the sum of the dimension weights equals d.

Then, the individual dimensions are identified. Denote the critical value of a dimension by k, $\sum c_{i}$ denotes the sum of the deprivation dimension weights of individual i, and compare it with the value k, obtain the matrix of the number of deprived individuals $P_{k}$, when $c_{j}\geq k$, $P_{k}(y_{i};z)=1$, when $c_{j}<k$, $P_{k}(y_{i};z)=0$.

A multidimensional poverty measure based on the modified FGT proposed by Alkire and Foster’s [46] Multidimensional Poverty Index $M_{0}=\mu (g(k))=HA$, where g(k) is the new matrix obtained by replacing all the row elements of the deprivation matrix with 0 for all non-deprived individuals, and μ is the average of the elements in g(k). Additionally, $M_{0}$ can finally be expressed as consisting of two components, H (incidence of poverty) and A (average deprivation share).

### 3.2. Methods

In order to more accurately analyze the impact of farmland transfer on rural elderly poverty, the analysis controls for individual elderly characteristics and household characteristics. The model is set up as follows:

(3) $$poverty_{i}=\beta_{0}+\beta_{1}transfer_{i}+X_{i}^{\prime }\delta +\varepsilon_{i}$$

where i denotes survey respondents, $poverty_{i}$ is the explanatory variable and indicates whether survey respondent rural elderly i is in poverty; $transfer_{i}$ is the explanatory variable and indicates the existence of a transfer of agricultural land from the surveyed rural elderly i; $X_{i}$ is a set of control variables derived from the individual characteristics and household characteristics of the respondents; $\varepsilon_{i}$ is the random error term.

## 4. Data Collection and Analysis

### 4.1. Data Collection

The data used in this paper are from the rural sample of the China Family Panel Studies (CFPS). These data have been organized and implemented by the China Social Science Survey Centre of Peking University since 2010 and are tracked every two years, covering 25 provinces, municipalities, and autonomous regions across China. For sample selection, the latest published individual and household samples of the 2018 survey were used based on the research needs of this paper. Invalid and missing samples were excluded to obtain a final sample of 4585 rural elderly data, covering the household situation, economic status, living conditions, health status, and social welfare level of the rural elderly.

### 4.2. Indicator System Construction

The multidimensional relative poverty indicator system in this paper is constructed based on the multidimensional poverty indicator system. The multidimensional poverty dimensions selected globally generally include the Human Development Index (HDI), Human Poverty Index (HPI), and the Multidimensional Poverty Index (MPI), taking into account the particular characteristics of the birth and education years of the rural elderly group in China [47]. This paper selects three dimensions to measure multidimensional relative poverty—economic level, quality of life, and health status—excluding the education dimension. These three dimensions correspond to the three dimensions of poverty: economic poverty, welfare poverty, and capability poverty, with economic level corresponding to economic poverty, quality of life corresponding to welfare poverty, and health status corresponding to capability poverty. Based on the characteristics of the 2018 CFPS data, the dimensions were selected and indicators assigned as follows:

Economic level—the sample households’ 50% median per capita disposable income is chosen as the relative poverty threshold. In existing multidimensional poverty studies, the comparison between annual net per capita income and the threshold value (poverty line for the year) is usually chosen to determine the economic dimension of poverty. To upgrade the previous definition of absolute poverty in terms of economic level to the discrimination of relative poverty, and taking into account China’s national conditions, this study selects the reference indicator suggested by Vliet and Wang [48]. Considering 50% of the median per capita disposable income as the relative poverty line, below this level is assigned as 1, inferring relative economic poverty, and vice versa, a value of 0 is assigned [49,50].

Health status—the specific nature of the elderly population necessitates considering their health status when investigating poverty among the elderly. The commonly used domestic quality of life indicators (QWB) are demanding in terms of data. Subsequently, many scholars use self-rated health variables that are relatively easy to obtain [51,52]. The same self-assessed health variables are used in the current study, and two indicators are selected: physical health and mental loneliness, where physical health is based on self-assessed health status compared to peers. The frequency of “I feel lonely” is used, with more than one time in a week being assigned a value of 1, meaning that there is mental poverty, and the opposite being assigned a value of 0.

Quality of life—two indicators are selected in this study: fuel for living and drinking water. Specifically, clean fuels, such as natural gas, gas, induction cookers, and solar energy, which are commonly used by households, are used as an indicator of poverty in terms of fuel for living. The use of clean water is used as a measure of poverty in terms of drinking water. The dimensions and indicators selected and the thresholds and weights for the multidimensional poverty measure in this paper are detailed in Table 1.

This study is based on the 2018 China Family Panel Studies (CFPS) data with the multidimensional relative indicator system shown in Table 1 and uses the AF multidimensional poverty measurement method introduced previously. The multidimensional relative poverty index of rural older people (see Table 2) is assessed, defining rural older people with two or more dimensions of poverty as being in multidimensional relative poverty. Rural older people with two or more dimensions of poverty are defined as being in multidimensional relative poverty, and those with no poverty or only one dimension of poverty as not being in multidimensional relative poverty.

Table 2 shows that when K = 1, i.e., when there is at least one dimension of poverty among the rural elderly in the sample, the incidence of poverty is 86.65%, which indicates that the majority of the rural elderly have a poverty issue, with an average poverty deprivation value of 0.4166, and the Multidimensional Poverty Index is 0.3610. When K = 2, i.e., when there are at least two dimensions of poverty among the rural elderly in the sample, the incidence of poverty is 37.71%. When K = 3, the incidence of poverty is 8.35%, indicating that fewer rural older people have all three dimensions of poverty. At 8.35%, the average deprivation value is 0.8786, and the Multidimensional Poverty Index is 0.2378. It can be seen that as the number of dimensions increase, the incidence of poverty decreases, and the average deprivation value rises. At the same time, as the incidence of poverty decreases much more than the average deprivation share rises, the MPI eventually decreases.

### 4.3. Data Analysis

This paper examines the impact of the transfer of agricultural land on the multidimensional relative poverty of rural elderly people. The dependent variable is the multidimensional relative poverty of rural elderly people. Rural elderly people with two or more dimensions of poverty are defined as being in a state of multidimensional relative poverty. The selected control variables are mainly derived from the individual characteristics, household characteristics, and social welfare characteristics of the rural elderly, including age, gender, education level, marital status, number of children, intergenerational support, neighborhood relationship, pension insurance, and medical insurance, and the calculation method and descriptive statistics are shown in Table 3.

## 5. Analysis of Empirical Results

### 5.1. Analysis of the Impact of Agricultural Land Transfers on a Single Poverty Indicator among Rural Older People

From the previous theoretical analysis, the impact of farmland transfer on rural old-age poverty is more complex and may have a two-way impact on the same dimensional indicator of poverty. It may also have different impacts on alternative dimensional indicators of poverty. To test this claim, research hypothesis H1, this study performs Logit regression estimation with each single poverty indicator in Table 1 as the explanatory variable in Equation (3). Before conducting the model estimation, due to the possible cointegration problem among the variables, this paper adopts the variance inflation factor method to conduct multiple cointegration tests on all independent variables. The results show that the VIF values of all variables are less than 10, and there is no cointegration problem.

Table 4 presents the results of estimating the impact of the agricultural land transfer on each individual dimensional indicator of poverty among rural older people. **Models 1–5** represent the effects of agricultural land alienation on the incidence of poverty for each indicator of rural older people’s relative economic income, physical health, mental loneliness, fuel use, and drinking water. The regression results show that the transfer of agricultural land has a significantly negative effect on the relative poverty status of rural older people’s per capita household income, fuel use poverty, and drinking water poverty incidence. The data also show a positive relationship between physical health poverty and mental loneliness among older people, but the regression results do not pass significance. Specifically, the agricultural land transfer effectively alleviates the economic poverty of rural older people, that is, the more land rural older people choose to transfer, the less likely they are to fall into economic poverty. The agricultural land transfer can effectively improve the quality of life of older people in rural areas and enable them to have better living conditions. In terms of the health status dimension, the agricultural land transfer is detrimental to the physical and mental health of rural older people, but its impact is very limited. It can be seen that land transfer has different effects on different dimensions of poverty among rural older people, while the effects on poverty indicators under the economic level and quality of life dimensions are more significant, and the research hypothesis H1 is verified.

### 5.2. Baseline Regression of the Impact of Agricultural Land Transfers on the Relative Multidimensional Poverty of Rural Older People

Based on the construction of the indicator system and the multidimensional relative poverty measure in the previous section, this paper examines the effect of farmland transfer on the relative multidimensional relative poverty status of the rural elderly (see Model 6) and further examines the effect of farmland transfer on the relative multidimensional poverty status of the rural elderly (see **Model 7**) by introducing three control variables on the individual characteristics, family characteristics, and social welfare characteristics of the elderly. The estimation results of **Model 6** in Table 5 show that farmland transfer out is statistically significant at the 1% level with a negative estimated coefficient. **Model 7**, after the introduction of control variables, shows that the transfer out of agricultural land has a negative effect on multidimensional relative poverty in rural old age at the 1% level. This means that, taken together, the transfer of agricultural land has a significant alleviating effect on rural old age poverty, and the research hypothesis H2 is verified.

### 5.3. Robustness Tests

**Figure 2:**
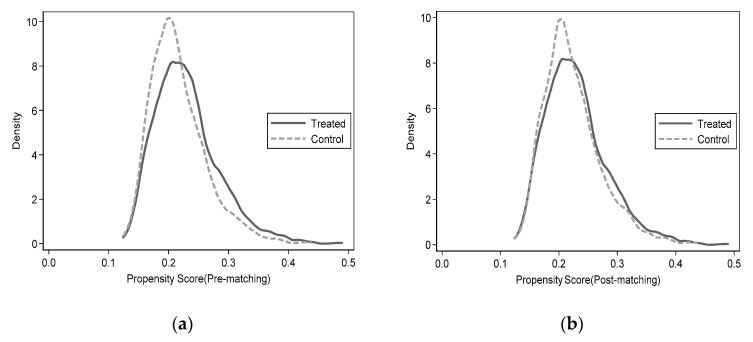
Kernel probability distribution before and after matching.

The previous study discusses the impact of agricultural land transfers on the multidimensional relative poverty of rural older people and includes variables on individual, household, and social welfare characteristics. However, the previous model may have endogeneity problems due to omitting other variables related to agricultural land transfers. In order to deal with the endogeneity problem and to check the robustness of the model estimates, the propensity value matching method (PSM) is used to match the samples before estimating the measures. Before using PSM for estimation, it is necessary to check whether the sample matching is reasonable and valid. This study uses the more commonly used kernel density function distribution of propensity scores to check the matching before and after matching the treatment group with the control group [52,53]. Figure 2a,b represents the kernel density distribution before and after matching farmland turn-out. From Figure 2, it can be seen that when the nearest neighbor matching method is used (other methods were also tried for matching in this study with similar results), the difference in the probability distribution of the propensity score matching values between the treatment and control groups decreases significantly. The propensity score intervals have a considerable overlap range, and most of the observed values are within the common range. This indicates that the matching method effectively reduces the differences in family characteristics between the treatment and control groups, suggesting that matching is more effective using the nearest neighbor matching method.

After checking for sample matches, a propensity value matching method (PSM) estimation will be carried out as follows: first, the probability of an older person falling into multidimensional relative poverty is estimated based on observable individual and household characteristics (age, gender, education level, marital status, number of children, intergenerational communication, neighborhood, pension, and health insurance of the older person are included in this study) and the propensity score is calculated, which can be expressed as:

(4) $$P(X_{i})=\mathrm{Pr}(F_{i}=1|X_{i})=\frac{\exp(\beta X_{i})}{1+\exp(\beta X_{i})}+\varepsilon$$

The binary dummy variable *F* in Equation (4) is expressed as the farmland transfer out characteristic, $X_{i}$ indicates the relevant influencing factor, β is the model’s coefficient, and ε is the random error. Then, find the older people whose farmland has not been transferred who have the most similar score to the propensity to transfer farmland as their counterfactual, then compare the difference in falling into multidimensional relative poverty between the two groups. Take the mean of the calculated differences to obtain the average effect of transferring farmland on rural older people falling into multidimensional relative poverty, which can be expressed as:

(5) $$ATT=E(Y_{i,1}|T_{i}=1)-E(Y_{i,0}|T_{i}=1)$$

This paper selected three matching methods, including neighbor, radius, and kernel, for the propensity value matching process. The feature variables changes, before and after matching the samples obtained after caliper matching (this paper also tried to match using other methods, and the changes were similar), are shown in Table 6. Based on existing studies, the absolute value of the standard deviation after matching is usually equal to 10 as the criterion for determining the matching effect. If the absolute value of the standard deviation after matching is less than 10, the matching effect is better. As can be seen from the data in Table 6, after matching, the standard deviations of the characteristic variables of both groups of samples were significantly reduced. The absolute value of the standard deviation was less than 10, which indicates that the difference between the mean values of each characteristic variable is minimal. The differences in characteristics between the samples were eliminated to a certain extent, and the matching effect was improved.

To ensure the robustness of the estimation results, the average treatment effects of the impact of farmland switching out on multidimensional relative poverty among rural older people was assessed using neighbor matching, caliper matching (Radius), and quadratic kernel matching, respectively. The mean treatment effect estimates in Table 7 show that the pre-matching effect of farmland transfers reduces poverty incidence among rural older people by 7.22% at the 1% significance level. When differences between the sample control and treatment groups are eliminated using the matching method, the mean net effect ATT ranges from 7.98% to 8.04%, meaning that the transfer of farmland reduces rural elderly multidimensional relative poverty by around 8%. This is increased compared to the regression coefficient before matching, suggesting that using ordinary Logit regression would underestimate the impact of farmland transfer on rural elderly multidimensional relative poverty. PSM estimates further supports the H2 hypothesis that farmland transfer reduces the probability of rural older people falling into poverty.

## 6. Discussion

The empirical results prove the two hypotheses proposed in this paper and pass the robustness test. From H1, as one of the basic means of production and production factors for agricultural development has a decisive influence on the livelihood ability of farmers, and with the aging of rural labor, the income-generating capacity of rural elderly people declines as their physical functions decline [54]. This is where land plays its role as a “bottom-up” function in securing the livelihoods of rural older people [55,56], and its multiple functions of production, security, and property are expressed more in terms of economic output and income [57]. When older people in rural areas choose to transfer their farmland, it is equivalent to discounting the value of the land, which directly translates the property income function of the land into economic income, thus contributing to a better quality of life [58]. There may be positive and negative bidirectional effects of agricultural land transfer on two indicators of poverty: physical health and mental loneliness, as described in the theoretical analysis, making the results insignificant [35,59]. As an important household decision, the main participants and direct beneficiaries of the outcome of agricultural land transfer are the elderly in the household, and thus, the combined effect of agricultural land transfer on poverty among rural elderly needs to be evaluated to inform decision-making behavior.

The empirical results from H2 show that land transfer out effectively alleviates multidimensional poverty among the rural elderly. In the absence of land transfer, land resources in rural China are allocated according to the number of household members, so when the number of household members is certain, the land resources owned by each rural household are scarce [60,61]. Given the scarcity of land resources, what are the reasons for rural older people to transfer their land holdings to secure their livelihoods? The reason for this phenomenon is that the rural social security system is significantly weaker than the urban social security system, both in absolute and relative terms, in the process of industrialization and urbanization, and the instability of family pensions due to the exodus of children and the miniaturization of family size, which makes the rural elderly rely to a greater extent on land income for their pension security [55]. The transfer of agricultural land, while preserving the subsidies of the elderly, highlights the property income function of land and is an important way to increase land income, which has a strong effect on alleviating the multidimensional poverty of the rural elderly.

## 7. Conclusions and Recommendations

In the realistic context of agricultural modernization development and comprehensive poverty eradication by 2020, this study analyses the impact of the agricultural land transfer on rural elderly poverty using Logit regression estimation and the propensity score matching method (PSM). The analysis is based on 2018 CFPS data. The study results show that (1) the current rural elderly poverty situation is more severe, with 86.65% of rural elderly people have single-dimensional poverty. More than half of the rural elderly have multidimensional relative poverty problems. (2) The transfer of agricultural land affects different dimensions of poverty among the rural elderly. Specifically, the transfer of agricultural land has a significantly adverse effect on the relative economic poverty, fuel poverty, and drinking water poverty of the rural elderly. The land transfer has a positive relationship with physical health poverty and mental health poverty of the elderly, but the regression results do not pass the significance test. (3) The transfer of agricultural land significantly impacts the incidence of multidimensional relative poverty among the rural elderly. The transfer of agricultural land reduced the incidence of multidimensional relative poverty among the rural elderly by approximately 8%, which can be regarded as a significant alleviating effect of the transfer of agricultural land on the comprehensive impact of poverty among the rural elderly.

These findings have important implications for the transfer of agricultural land and the alleviation of old-age poverty in rural areas. Specific policy implications from the results of this study can be outlined as: Firstly, to encourage the reasonable and effective transfer of agricultural land, while transferring agricultural land, it is essential to insist on the reversibility of land transfer, guarantee a reasonable level of land rent, and ensure a high level of commitment. Finally, a sound system of social security and care services for the elderly must be established in rural areas. Regarding rural labor exodus, the agricultural land transfer can alleviate rural elderly poverty; however, poverty alleviation is more reflected in the economic level and quality of life dimension. Given the current average contracted land area and land rent level in rural China, poverty alleviation in the economic and quality of life dimension may be very limited. “In order to make up for the current shortcomings of family pensions and land pensions, and to give the rural elderly a sense of security, it is necessary to speed up the establishment of a modern rural social security and care service system for the rural elderly”.

## Figures and Tables

**Table 1:** Dimension, index, threshold, and weight setting.

Dimensionality	Indicators	Threshold and Assignment	Weighting
Economic level	eco1: Household income per capita relative poverty status	A net household income per capita below 60% of the median household in the sample is assigned a value of 1, while the opposite is assigned a value of 0	1/1
Health status	hel1: Physical health self-assessment	Self-assessed health status of bad or very bad compared to peers is assigned a value of 1, while the opposite is assigned a value of 0	1/2
	hel2: Mental isolation status	1 for sometimes, often, or always feeling lonely, 0 for the opposite	1/2
Quality of life	lif1: Fuel use	No electricity, gas, natural gas, or solar energy is assigned a value of 1, while the opposite is assigned a value of 0	1/2
	lif2: Drinking water situation	1 if the main drinking water is not tap-water (including pure water, etc.), 0 if opposite is true	1/2

**Table 2:** Multidimensional relative poverty index of the rural elderly.

K-Value	Number of Multidimensional Poverty	Total Deprivation of Poverty	H (Incidence of Poverty) (%)	A (Average Deprivation Value of Poverty)	M (Multidimensional Poverty Index)
K = 1	3973	4966	86.65	0.4166	0.3610
K = 2	1729	3270.5	37.71	0.6305	0.2378
K = 3	383	1009.5	8.35	0.8786	0.0734

**Table 3:** Multidimensional relative poverty index of the rural elderly.

Variable Name	Meaning of Variables	Calculation Method	Mean	Standard Error
mrpi	Existence of multidimensional relative poverty	1 = presence of more than 1 dimension of poverty; 0 = no poverty or only a single dimension of poverty	0.3771	0.4847
ifzc	Whether to transfer out of agricultural land	1 = with transfer out; 0 = without transfer out	0.2190	0.4136
age	Age	Actual age of older people	67.6883	5.8182
gender	Gender	Gender of older people	0.5003	0.5001
education	Education level	1 = formally educated; 0 = illiterate	0.4408	0.4965
marriage	Marital status	1 = spouse (married); 2 unmarried, divorced, or widowed	0.8105	0.3920
children	Number of children	Number of surviving children of older people	2.3743	1.3407
intergenerational	Intergenerational communication	1 = able to see your child every day; 0 = not able to	0.4079	0.4915
neighborhood	Neighborhoods	1 = trust in neighbors of 5 or more; 0 = trust in neighbors of less than 5 (self-rating 10-point scale)	0.6807	0.4663
pension	Pensions	1 = with pensioner’s insurance; 0 = without	0.6659	0.4717
insurance	Medical insurance	1 = have medical insurance; 0 = no	0.9456	0.2266

**Table 4:** Empirical estimation results of rural poverty on rural elderly target poverty.

	Model 1	Model 2	Model 3	Model 4	Model 5
Variable Name	eco1	hel1	hel2	lif1	lif2
ifzc	−0.1853 **	0.0914	0.0728	−0.6920 ***	−0.4695 ***
	0.0872	0.0726	0.0768	0.0756	0.0787
age	0.0436 ***	0.0021	−0.0030	0.0037	0.0037
	0.0065	0.0057	0.0060	0.0058	0.0059
gender	0.1074	−0.4033 ***	−0.0802	0.1867 **	0.0013
	0.0762	0.0647	0.0689	0.0659	0.0671
education	−0.3093 ***	−0.1208 *	−0.2762 ***	−0.5024 ***	−0.0319
	0.0775	0.0651	0.0696	0.0664	0.0676
marriage	−0.0321	-0.1020	−1.0775 ***	0.1611 *	0.1092
	0.0939	0.0817	0.0841	0.0830	0.0856
children	0.0914 ***	0.0264	0.0207	0.0527 **	0.0524 **
	0.0266	0.0236	0.0249	0.0239	0.0244
intergenerational	−0.4105 ***	−0.1493 **	−0.4705 ***	−0.2345 ***	0.0035
	0.0752	0.0626	0.0678	0.0634	0.0646
neighborhood	−0.1855 **	−0.2680 ***	−0.1679 **	−0.1691 ***	−0.0864
	0.0744	0.0643	0.0678	0.0651	0.0663
pension	0.0336	−0.0352	0.0920	0.2609 ***	−0.1296 **
	0.0760	0.0642	0.0687	0.0652	0.0660
insurance	−0.3659 **	−0.0408	−0.0363	−0.1684	−0.3630 ***
	0.1450	0.1330	0.1401	0.1347	0.1335
constant	−3.5932 ***	0.3441	0.8522 *	−0.1230	−0.4235
	0.4764	0.4187	0.4431	0.4241	0.4317
R-squared	0.0274	0.0147	0.0460	0.0316	0.0098

Note: ***, **, * denote coefficients of explanatory variables significant at the 1%, 5%, and 10% levels, respectively.

**Table 5:** Empirical estimates of the relative multidimensional poverty of the rural elderly in rural land transfer.

Variable Name	Model 6		Model 7	
	Coefficient	Standard Error	Coefficient	Standard Error
ifzc	−0.3154 ***	0.0758	−0.3767 ***	0.0780
age			0.0243 ***	0.0059
gender			−0.0394	0.0675
education			−0.4294 ***	0.0684
marriage			−0.2620 ***	0.0836
children			0.0896 ***	0.0243
intergenerational			−0.4104 ***	0.0661
neighborhood			−0.2733 ***	0.0663
pension			0.0346	0.0672
insurance			−0.3174 **	0.1351
constant	−0.4351 ***	0.0342	−1.2469 ***	0.4307
R-squared	0.0029		0.0322	

Note: ***, ** denote coefficients of explanatory variables significant at the 1% and 5% levels, respectively.

**Table 6:** Changes in feature variables before and after sample matching (radius).

		Mean		Standard Deviation (%)	Deviation Reduction (%)	*t*-Test	
Variables	Sample	Interactive	Controls			t-Value	*p*-Value
age	Before matching	68.5230	67.4540	18.2	96.7	5.16	0.000
	After matching	68.5000	68.5350	−0.6		−0.13	0.898
gender	Before matching	0.4811	0.5057	−4.9	95.3	−1.38	0.168
	After matching	0.4816	0.4804	−0.2		0.05	0.959
education	Before matching	0.4721	0.4320	8.1	89.3	2.26	0.024
	After matching	0.4716	0.4673	0.9		0.19	0.848
marriage	Before matching	0.7610	0.8244	−15.7	92.1	−4.54	0.000
	After matching	0.7617	0.7567	1.2		0.26	0.792
children	Before matching	2.4522	2.3524	7.3	86.5	2.08	0.037
	After matching	2.4516	2.4382	1.0		0.21	0.831
intergenerational	Before matching	0.3835	0.4147	−6.4	81.2	−1.78	0.075
	After matching	0.3839	0.3897	−1.2		−0.27	0.787
neighborhood	Before matching	0.6942	0.6769	3.7	98.6	1.04	0.298
	After matching	0.6939	0.6937	0.1		0.01	0.991
pension	Before matching	0.6653	0.6660	−0.1	6.3	−0.04	0.968
	After matching	0.6650	0.6656	−0.1		−0.03	0.976
insurance	Before matching	0.9373	0.9481	−4.6	88.3	−1.34	0.182
	After matching	0.9372	0.9385	−0.5		−0.12	0.906

**Table 7:** Estimated results of average treatment effect.

Matching Method	Treated	Controls	Difference	S.E.	t-Value
Neighbor	0.3200	0.3998	−0.0798	0.0190	−4.19 ***
Radius	0.3200	0.4004	−0.0804	0.0171	−4.71 ***
Kernel	0.3200	0.3999	−0.0799	0.0169	−4.71 ***

Note: *** denote coefficients of explanatory variables significant at the 1% levels, respectively.

## Data Availability

The data are available online at http://www.isss.pku.edu.cn/cfps (accessed on 10 October 2020).
